# Detection of hepatitis B virus isolates with mutations associated with immune escape mutants among pregnant women in Ibadan, southwestern Nigeria

**DOI:** 10.1186/s40064-015-0813-1

**Published:** 2015-02-01

**Authors:** Temitope Oluwasegun Cephas Faleye, Moses Olubusuyi Adewumi, Ijeoma Maryjoy Ifeorah, Ewean Chukwuma Omoruyi, Solomon Adeleye Bakarey, Adegboyega Akere, Funmilola Awokunle, Hannah Opeyemi Ajibola, Deborah Oluwaseyi Makanjuola, Johnson Adekunle Adeniji

**Affiliations:** Department of Virology, College of Medicine, University of Ibadan, Ibadan, Oyo State Nigeria; Department of Surgery, College of Medicine, University of Ibadan, Ibadan, Oyo State Nigeria; Department of Medicine, College of Medicine, University of Ibadan, Ibadan, Oyo State Nigeria; Institute of Child Health, College of Medicine, University of Ibadan, Ibadan, Oyo State Nigeria; Institute for Advanced Medical Research and Training, College of Medicine, University of Ibadan, Ibadan, Oyo State Nigeria; WHO National Polio Laboratory, University of Ibadan, Ibadan, Oyo State Nigeria; Department of Microbiology, Faculty of Science, Ekiti State University, Ado Ekiti, Ekiti State Nigeria; Department of Science Laboratory Technology, Faculty of Science, Ekiti State University, Ado Ekiti, Ekiti State Nigeria

**Keywords:** HBV, Immune escape mutant, Nigeria, Pregnant women, Vaccine escape mutant

## Abstract

Perinatal transmission of hepatitis B virus (HBV) and its associated immune escape mutants (IEMs), is the major vehicle through which a population of chronically infected people who serve as infectious HBV reservoirs is maintained in communities. Therefore, to assess the risk of perinatal transmission, 272 pregnant women attending ante-natal clinics in Ibadan metropolis, southwestern, Nigeria, were screened for HBsAg using ELISA technique. Samples positive for HBsAg were subjected to HBV DNA detection by PCR amplification of the *S-*gene and amplicon sequencing. Isolates were genotyped and subtyped using a combination of molecular techniques.

Fifteen (5.5%) of the pregnant women were positive for HBsAg of which HBV DNA was detected in seven. Five of the isolates were typed as genotype E subtype ayw4 using amino acid residues at positions 122, 127, 134 and 160. Another could only be typed as genotype E subtype ayw4 by further phylogenetic analysis. The remaining one isolate did not belong to any of genotypes A – H. Three of the HBV isolates including the untypable, had mutations in the ‘a’ determinant associated with IEMs.

This study confirms the endemicity of HBV, the risk of perinatal transmission and the circulation of genotype E subtype ayw4 in Nigeria. It further demonstrates the presence of IEMs in Nigeria.

## Introduction

Hepatitis B virus (HBV) belongs to the genus *orthohepadnavirus* in the family *Hepadnaviridae*. HBV is an enveloped virus with a diameter of ~42 nm. Within the core of the virus is a protein-linked, ~3.2 kb DNA genome that is partly double stranded. The HBV genome has four open reading frames (ORFs) (X, S, P and C) with the X and C ORFs partially overlapping the P ORF which also has the S ORF within it but in a different reading frame. Genotypes A - H of HBV have been described (Schaefer 2007) with members of a genotype not differing by more than 8% of their genome (Okamoto *et al.*^1988^). Sub-genotypes have also been described with members not differing by more than 4% of their genome (Norder *et al.*^2004^).

Hepatitis B virus (HBV) infection is one of the top 10 viral infections globally (Perz *et al.*^2006^). Serologic evidence of past or present HBV infection has been shown in approximately one-third of the world’s population (i.e., over 2 billion people) (World Health Organization 2009). Infection with HBV could result in outcomes ranging from an acute, self-limiting disease through chronic hepatitis B (CHB) to cirrhosis and hepatocellular carcinoma (HCC) (Gerlich 2013). Furthermore, about 360 million people globally are chronically infected with HBV (World Health Organization 2009).

About 90% of children who get infected perinatally become chronic HBV carriers (Beasley *et al.*^1981^; Chang 2007). Hence, vertical transmission of HBV is a major vehicle through which a reservoir of infectious HBV is maintained in populations (Beasley *et al.*^1981^; Chang 2007). This can and is being addressed by identifying and treating HBV infected pregnant women and administering both passive (Beasley *et al.*^1981^) and active (Poovorawan *et al.*^1989^; Lavanchy 2012) immunization to their offspring at birth. However, there have been cases in which children or adults who have been vaccinated against HBV and have serologic correlates of HBV immunity get re-infected with HBV (Carman *et al.*^1990^; Harrison *et al.*^1991^; Fujii *et al.*^1992^; Theamboonlers *et al.*^2001^). The HBV isolates recovered from such cases are usually referred to as Immune Escape Mutants (IEMs) (Carman *et al.*^1990^; Lada *et al.*^2006^; Ramezani *et al.*^2013^).

Immune escape mutants (IEMs) were first described in Italy in 1990 (Carman *et al.*^1990^). The development of IEMs has been ascribed to the fact that HBV has both an RNA phase in its replication cycle and a polymerase without ‘proof-reading’ ability, and as such has an error rate that is close to that of RNA viruses (Orito *et al.*^1989^; Mizokami and Orito 1999). Consequently, mutants develop spontaneously and can be selected for by anti-HBs and the use of nucleoside/nucleotide analogue classes of antiviral drugs (Cento *et al.*^2013^).

Several studies have reported the presence and circulation of IEMs (Carman *et al.*^1990^; Harrison *et al.*^1991^; Fujii *et al.*^1992^; Theamboonlers *et al.*^2001^; Forbi *et al.*^2013^) but not in Nigeria. Since HBV vaccination was added to the National Immunization programme over ten years ago (WHO 2005a; Sadoh and Eregie 2008) and nucleoside/nucleotide analogue classes of antiviral drugs are being used by the HIV and/or HBV infected population in the country (WHO 2005b), this study was designed to investigate the possible emergence and circulation of IEMs in pregnant women in Nigeria. Here we report, for the first time, the presence of HBV IEMs in Nigeria.

## Material and methods

### Study location

This study was carried out among pregnant women attending two different ante-natal clinics in Ibadan, southwestern Nigeria. The two hospitals were selected to facilitate true representation of the population in the study. The first being the University College Hospital (UCH); a tertiary health care facility of the University of Ibadan. The second hospital is Ade-Oyo State Hospital (ASH); a secondary health care facility located in a central, densely populated part of the city. Attendees in both ante-natal clinics are majorly residents in the city.

### Enrolment of patients

Consenting ante-natal clinic attendees were enrolled from the two selected hospitals described above. Enrolment took place between September 2012 and June, 2013. During the period, a total of 813 and 1686 ante-natal clinic attendees were enrolled at the ASH and UCH respectively. Short presentation on HBV prevalence and prevention was given during each visit to the ante-natal clinics. Thereafter, consenting clinic attendees were enrolled for the study. Demographic and other relevant information were retrieved from the study participants using a structured questionnaire. Afterwards, blood sample was collected from each of the 272 {median age = 31.07 years, age range = 17–43 years (NGRAD: n = 90; age range = 19–42 years; NGRUC: n = 182; age range = 17–43 years)} consenting subjects enrolled at the point of registration and routine examination for ante-natal clinic. Ethical approvals for the study were granted by the UI/UCH Ethics Committee (UI/EC/11/0058) and Ministry of Health (AD3/479/349).

### Sample collection

Five milliliters of blood was collected from each pregnant woman by venepuncture. The blood sample was then dispensed into an appropriately labeled sterile container without any preservative or anticoagulant. Subsequently, the samples were transported to the laboratory at about 4-8°C in a cooler with frozen ice packs. Serum was separated from other blood components by low-speed centrifugation at 500 g for 5 minutes and subsequently removed using a sterile disposable pipette. Two aliquots of serum were made per sample in labeled sterile cryovials which were stored at −20°C until ready for analysis. Laboratory analysis was carried out in the Department of Virology, and the Institute for Advanced Medical Research and Training, College of Medicine, University of Ibadan, Ibadan, Nigeria.

### HBsAg ELISA screen

All the 272 samples were subjected to HBsAg specific Enzyme Linked Immunosorbent Assay (ELISA) using the HBsAg detection ELISA kit (Diagnostic Automation/Cortez Diagnostic, California, USA). The assay was performed according to manufacturer’s instructions. The optical density was read using the Emax endpoint ELISA microplate reader (Molecular Devices, California, USA) and the result was interpreted according to the manufacturer’s instructions.

### DNA extraction and HBsAg specific Polymerase Chain Reaction (PCR)

DNA was extracted using the QIAGEN DNA extraction kit (Qiagen, Hilden, Germany) according to the manufacturer’s instructions. Subsequently, a nested PCR assay targeting a ~408 bp stretch within the S ORF was used to detect HBV DNA. First round primers were HBV_S1F 5-CTAGGACCCCTGCTCGTGTT-3, and HBV_S1R 5-CGAACCACTGAACAAATGGCACT-3, while second round primers were HBV_SNF 5-GTTGACAAGAATCCTCACAATACC-3 and HBV_SNR 5-GAGGCCCACTCCCATA-3 (Forbi *et al.*^2013^). Primers were made in 25 μM concentrations and two microliter of each of the primers was added to a 50 μL reaction containing 10 μL of Red load Taq (Jena Bioscience, Jena, Germany), 4 μL of DNA and 32 μL of RNase free water. Thermal cycling was done in a Veriti Thermalcycler (Applied Biosystems, California, USA.) as follows; 94°C for 3 minutes followed by 45 cycles of 94°C for 30 seconds, 55°C for 60 seconds and 70°C for 40 seconds with ramp of 40% from 55°C to 70°C. This was then followed by 72°C for 7 minutes and held at 4°C till terminated. Reaction conditions were the same for both first and second round PCR except that DNA extract from the sample was used as template for first round PCR while first round PCR product was used as template for second round PCR. Finally, PCR products were resolved on 2% agarose gels stained with ethidium bromide and viewed using a UV transilluminator.

### Amplicon sequencing

The PCR reactions that were positive by having the required amplicon size were shipped to Macrogen Inc, Seoul, South Korea, for amplicon purification and BigDye chemistry sequencing. Sequencing was done using primers HBV_SNF and HBV_SNR.

### Phylogenetic analysis

Amino acid residues at positions 122, 127, 134 and 160 were initially used to determine HBV genotypes and serotypes (Forbi *et al.*^2013^). Subsequently, HBsAg reference sequences were retrieved from the HBV database (http://hbvdb.ibcp.fr/HBVdb/) and aligned using the CLUSTAL W program in MEGA 5 software with default settings (Tamura *et al.*^2011^). Afterwards, a neighbor-joining tree was constructed using MEGA 5 software with the Kimura-2 parameter model (Kimura 1980) and 1,000 bootstrap replicates. Furthermore, pairwise distance of the HBsAg sequences were estimated using MEGA 5 software with Kimura-2 parameter model (Kimura 1980). The accession numbers of sequences retrieved from HBV database for phylogenetic analysis are indicated in the sequence names on the phylograms.

### Nucleotide sequence accession numbers

The sequences reported in this study have been submitted to GenBank Nucleotide Sequence Database under the accession numbers KM225621 - KM225627.

## Results

### HBsAg ELISA screen, DNA extraction and Surface/Pol gene specific Polymerase Chain Reaction

A total of 15 (5.5%) of the 272 samples were positive for HBsAg. DNA was extracted from all the 15 samples that were positive for the HBsAg ELISA screen, and subjected to HBsAg specific PCR. Despite repeated attempts the ~408 bp amplicon within the S ORF was successfully amplified in only 7 (46.7%) of the 15 HBsAg positive samples.

### Phylogenetic analysis

**Figure 1:**
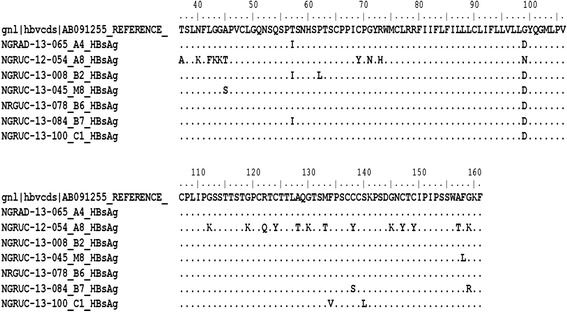
**Alignment of amino acid residues of the seven isolates sequenced in this study against a reference genotype E strain.** Of particular interest are isolates NGRUC-12-054-A8-HBsAg, NGRUC-13-084-B7-HBsAg and NGRUC-13-100-C1-HBsAg with mutations in the ‘a’ determinant (s122 to s160).

**Figure 2:**
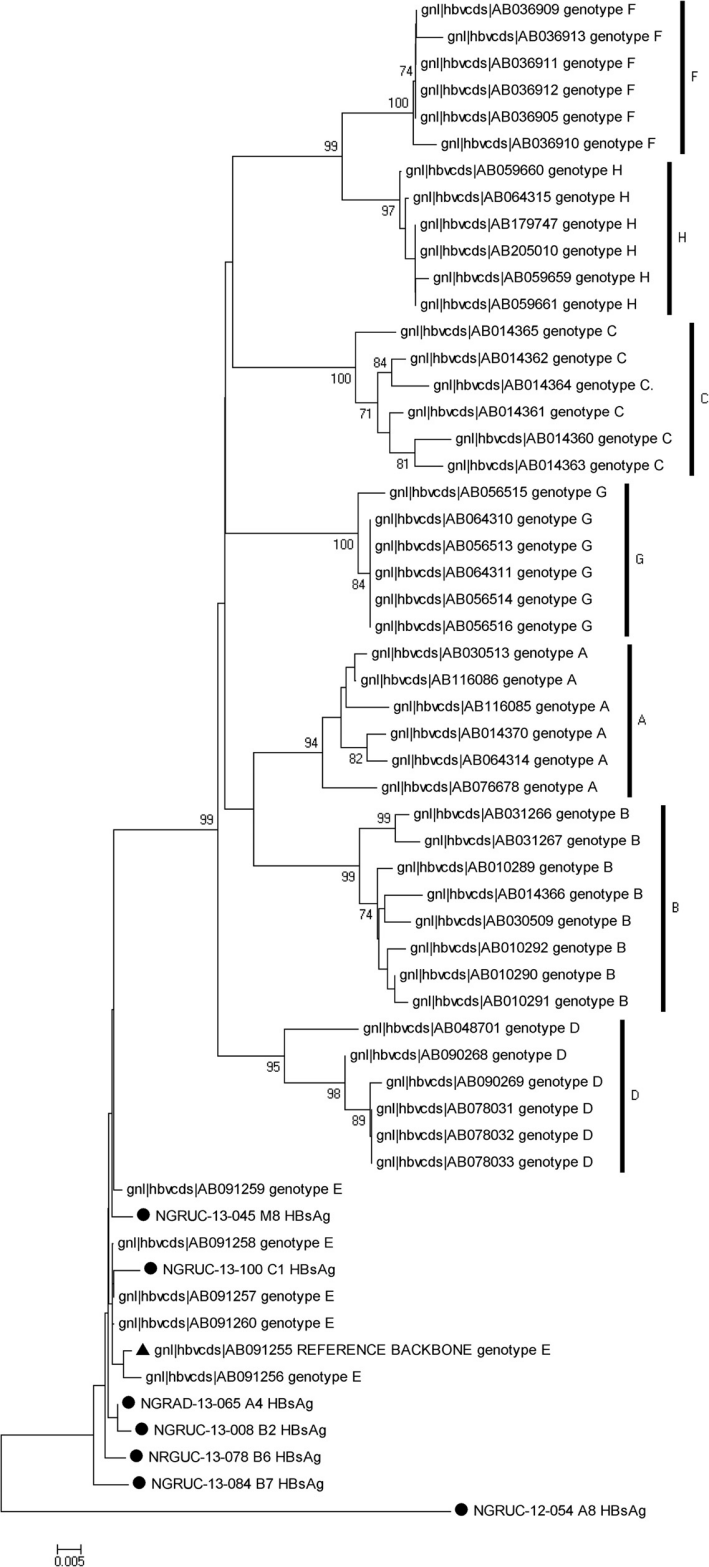
**Phylogenetic relationship of recovered HBV isolates.** The phylogram is based on an alignment of the partial HBsAg sequences. The newly sequenced strains are highlighted with black circle, while a genotype E reference strain is highlighted with a black triangle. The GenBank accession number of the strains are indicated in the tree. Bootstrap values are indicated if ≥ 70%.

Based on the amino acid residues at positions s122, s127, s134 and s160 (Forbi *et al.*^2013^), five of the seven sequenced isolates belonged to genotype E and serotype ayw4 (Table 1). The remaining two HBV isolates could not be typed using this classification scheme because isolate NGRUC-12-054-A8-HBsAg had sR122Q substitution and isolate NGRUC-13-100-C1-HBsAg had sF134V substitution (Figure 1). To classify the two untypable HBV isolates, a phylogram was constructed using the sequences isolated in this study alongside reference HBV sequences of genotypes A–H from the HBV database (http://hbvdb.ibcp.fr/HBVdb/). The phylogram (Figure 2) showed that, just like the previously classified five isolates, isolate NGRUC-13-100-C1-HBsAg belonged to genotype E, ayw4. Furthermore, isolate NGRUC-13-100-C1-HBsAg is only 1.1% divergent (Table 2) from a reference genotype E, ayw4 strain (AB091255). Hence, confirming its membership in this group.

**Table 1:** **Serotype and genotype of sequenced HBV isolates**

**S/N**	**Sample ID**	**Amino acid residues at positions within the surface antigen for serotype determination**				**Serotype**	**Genotype**
		**s122**	**s127**	**s134**	**s160**		
1	NGRAD-13-065-A4-HBsAg	R	L	F	K	ayw4	E
2	NGRUC-12-054-A8-HBsAg	Q	L	F	K	Untypable	Untypable
3	NGRUC-13-008-B2-HBsAg	R	L	F	K	ayw4	E
4	NGRUC-13-045-M8-HBsAg	R	L	F	K	ayw4	E
5	NGRUC-13-078-B6-HBsAg	R	L	F	K	ayw4	E
6	NGRUC-13-084-B7-HBsAg	R	L	F	K	ayw4	E
7	NGRUC-13-100-C1-HBsAg	R	L	V	K	Untypable	Untypable

**Table 2:** **Comparison of reference sequences gnl|hbvcds|AB091255 genotype E to all isolates recovered in this study**

**Species 1**	**Species 2**	**Dist %**	**Siml %**
gnl|hbvcds|AB091255 REFERENCE BACKBONE genotype E	NGRAD-13-065 A4 HBsAg	0.8	99.2
gnl|hbvcds|AB091255 REFERENCE BACKBONE genotype E	NGRUC-12-054 A8 HBsAg	12.5	87.5
gnl|hbvcds|AB091255 REFERENCE BACKBONE genotype E	NGRUC-13-008 B2 HBsAg	1.1	98.9
gnl|hbvcds|AB091255 REFERENCE BACKBONE genotype E	NGRUC-13-045 M8 HBsAg	1.1	98.9
gnl|hbvcds|AB091255 REFERENCE BACKBONE genotype E	NRGUC-13-078 B6 HBsAg	1.1	98.9
gnl|hbvcds|AB091255 REFERENCE BACKBONE genotype E	NGRUC-13-084 B7 HBsAg	1.6	98.4
gnl|hbvcds|AB091255 REFERENCE BACKBONE genotype E	NGRUC-13-100 C1 HBsAg	1.1	98.9
gnl|hbvcds|AB091255 REFERENCE BACKBONE genotype E	gnl|hbvcds|AB091260 genotype E	0.5	99.5

The phylogram (Figure 2) also showed that isolate NGRUC-12-054-A8-HBsAg, did not belong to any of genotypes A – H. Rather, it stood apart as an outgroup. Similarity matrix (Table 3) also showed that it is between 12.0 and 18.9% divergent from genotypes A – H and as such does not belong to any of the genotypes. It is however most closely related to genotype E at 12.0%. The Major Hydrophilic Region (MHR; s122 – s160) of isolate NGRUC-12-054-A8-HBsAg showed an abundance of substitutions which included; sG112K, sG119K, sR122Q, sC124Y, sA128T, sG130K, sM133T, sC138Y, sG145K, sC147Y, sC149Y, sA157T and sG159K (Figure 1).

**Table 3:** **Comparison of isolate NGRUC-12-054 A8 HBsAg to all isolates recovered in this study and reference sequences from genotypes A to H**

**Species 1**	**Species 2**	**Dist %**	**Siml %**
NGRUC-12-054 A8 HbsAg	NGRUC-13-008 B2 HBsAg	12.6	87.4
NGRUC-12-054 A8 HbsAg	NGRUC-13-045 M8 HBsAg	12.2	87.8
NGRUC-12-054 A8 HbsAg	NRGUC-13-078 B6 HBsAg	12.0	88.0
NGRUC-12-054 A8 HbsAg	NGRUC-13-084 B7 HBsAg	12.2	87.8
NGRUC-12-054 A8 HbsAg	NGRUC-13-100 C1 HBsAg	12.6	87.4
NGRUC-12-054 A8 HbsAg	gnl|hbvcds|AB116085 genotype A	18.2	81.8
NGRUC-12-054 A8 HbsAg	gnl|hbvcds|AB116086 genotype A	16.8	83.2
NGRUC-12-054 A8 HbsAg	gnl|hbvcds|AB031266 genotype B	18.2	81.8
NGRUC-12-054 A8 HbsAg	gnl|hbvcds|AB031267 genotype B	17.8	82.2
NGRUC-12-054 A8 HbsAg	gnl|hbvcds|AB014364 genotype C.	18.9	81.1
NGRUC-12-054 A8 HbsAg	gnl|hbvcds|AB014365 genotype C	17.2	82.8
NGRUC-12-054 A8 HbsAg	gnl|hbvcds|AB090268 genotype D	17.3	82.7
NGRUC-12-054 A8 HbsAg	gnl|hbvcds|AB090269 genotype D	18.4	81.6
NGRUC-12-054 A8 HbsAg	gnl|hbvcds|AB091260 genotype E	12.0	88.0
NGRUC-12-054 A8 HbsAg	gnl|hbvcds|AB036912 genotype F	18.2	81.8
NGRUC-12-054 A8 HbsAg	gnl|hbvcds|AB036913 genotype F	18.9	81.1
NGRUC-12-054 A8 HbsAg	gnl|hbvcds|AB064310 genotype G	17.6	82.4
NGRUC-12-054 A8 HbsAg	gnl|hbvcds|AB064311 genotype G	17.6	82.4
NGRUC-12-054 A8 HbsAg	gnl|hbvcds|AB179747 genotype H	18.2	81.8
NGRUC-12-054 A8 HbsAg	gnl|hbvcds|AB205010 genotype H	18.2	81.8
NGRUC-12-054 A8 HbsAg	NGRAD-12-065 A4 HBsAg	12.2	87.8
NGRUC-12-054 A8 HbsAg	Gnl|hbvcds|AB091255 reference backbone genotype E	12.5	87.5

Other isolates asides NGRUC-12-054-A8-HBsAg also have significant substitutions within the MHR. These included, isolates NGRUC-13-084-B7-HBsAg (sC138S and sG159R) and NGRUC-13-100-C1-HBsAg (sF134V and sS140L) (Figure 1).

## Discussion

### Mutations in the S gene and vaccine escape mutants

The results of this study showed the presence of G145K in one of the HBV isolates (NGRUC-12-054-A8-HBsAg) detected. This substitution alongside G145R have been associated with immune escape mutants (IEMs) (Carman *et al.*^1990^, ^1995^; Karthigesu *et al.*^1994^; Grethe *et al.*^1998^; Lada *et al.*^2006^). This study therefore reports for the first time the presence of IEMs in Nigeria and particularly in a pregnant woman.

Asides the G145K substitution, NGRUC-12-054-A8-HBsAg had other substitutions that might work synergistically to enhance the immune escape phenotype predicted. For example, the substitution of cysteine residues at positions s124, s138, s147 and s149 with tyrosine (Figure 1) will abolish the formation of the disulphide bonds predicted to stabilise the double-loop structure of the HBsAg extravirion region (Prange and Streeck 1995). *In-silico* structure prediction studies (unpublished data) suggested that the three dimensional conformation of the extravirion loop of this isolate is different from that of the wild-type genotype E, ayw4 isolate. In addition, instead of being extravirion, the loop is embedded in the polar head-groups of the extravirion leaflet of the virion membrane. This buttresses the fact that this isolate might indeed be an IEM. Similar structural prediction results were obtained for isolates NGRUC-13-084-B7-HBsAg and NGRUC-13-100-C1-HBsAg which also have substitutions between residues s133 and s144 (unpublished data).

The results of this study showed that 3 out of the 7 isolates (NGRUC-12-054-A8-HBsAg, NGRUC-13-084-B7-HBsAg and NGRUC-13-100-C1-HBsAg) sequenced in this study had mutations that have been associated with immune escape mutants (IEMs) (Carman *et al.*^1990^, ^1995^; Karthigesu *et al.*^1994^; Grethe *et al.*^1998^; Lada *et al.*^2006^; Forbi *et al.*^2013^). This may imply considerable rate of IEM emergence. However, the ecological system necessary to create the adequate amount of evolutionary pressure necessary to select for HBV IEMs at such high rate is present in Nigeria. Factors necessary to provide such selective pressure include spontaneous errors by HBV polymerase, the host immune response to natural HBV infection, active and passive immunization, and use of nucleoside/nucleotide analogue antiviral drugs by HIV and/or HBV infected individuals (Cento *et al.*^2013^). The first two factors are intrinsic to HBV and humans, and are consequently always present while, the last two have been in place in Nigeria for over ten years (WHO 2005a, b; Sadoh and Eregie 2008;). This therefore shows that the conditions had always been present for HBV IEMs to emerge.

The HBV IEMs characterized in this study did not share mutations in the ‘a’ determinant (Figure 1). This shows that the IEMs must have emerged as a result of independent events. This finding has two implications. Firstly, it corroborates the fact that the conditions were perfect for HBV IEM emergence in Nigeria, and secondly, it shows that HBV immune escape mutants can evolve over a sequence and conformational space. However, as a result of the small number of isolates recovered in this study, it is difficult to extrapolate as regards the circulation of these mutants. What is obvious is that the results of this study do not provide evidence to support circulation of these mutants in the region and this is corroborated by the fact that none of the previous studies in the region ever reported detection of HBV IEMs (Odemuyiwa *et al.*^2001^; Forbi *et al.*^2010^).

It is pertinent to note that, despite repeated attempts the ~408 bp amplicon within the S ORF could not be amplified in 8 (53.3%) of the 15 HBsAg positive samples. This could be due to low viral load, primer mismatch as a result of point mutations or virus strains being of recombinant origin. However, effort is underway to further characterize the isolates using sequence independent strategies.

### HBV genotypes circulating in Nigeria and the classification scheme

The circulation in Nigeria of HBV genotype E, serotype ayw4 was documented in this study. This confirms previous reports (Odemuyiwa *et al.*^2001^; Forbi *et al.*^2010^). Furthermore, the findings of this study confirm the inclusion of Nigeria in the HBV genotype E crescent as previously described (Odemuyiwa *et al.*^2001^; Mulders *et al.*^2004^; Olinger *et al.*^2006^; Andernach *et al.*^2009^; Forbi *et al.*^2010^). However, the use of amino acid residues at positions s122, s127, s134 and s160, though useful for classifying HBV isolates, obviously has its limitations. For example, of the six HBV genotype E, serotype ayw4 isolates sequenced in this study, five could be typed as such using this algorithm. This algorithm failed to identify the last isolate because of an sF134V substitution. Had it not been for phylograms and similarity matrices one of the isolates would have gone untyped. It might therefore be necessary to combine methods when identifying HBV isolates. Also, the inclusion of valine as one of the amino acid residues that could be present at position 134 of HBV genotype E, ayw4 should be considered.

### HBsAg prevalence in pregnant women

In this study, HBsAg prevalence of 5.5% was observed among pregnant women in southwestern Nigeria. This is similar to a prevalence of 6% found in south-south Nigeria (Alegbeleye *et al.*^2013^) but different from higher prevalence (>8%) reported in other parts of the country (Ndams *et al.*^2008^; Olokoba *et al.*^2011^). The reasons for this variation is not clear, though several reasons have been suggested (Alegbeleye *et al.*^2013^). Given HBsAg prevalence of 5.5% (as the lower limit) among pregnant women in Nigeria, an annual birth cohort of 7,310,490 in Nigeria (Gavi Alliance 2014), and a <5% risk of developing CHB in children that acquire HBV perinatally from HBsAg positive but HBeAg negative mothers (Hsu *et al.*^2004^; Chang 2007). This implies that about 20,104 children on the average might be developing CHB annually in Nigeria.

As observed in Taiwan (Su *et al.*^2012^) and Thailand (Poovorawan *et al.*^2011^), with the incorporation of HBV vaccine into the National Immunization Programme and significant vaccine coverage, the number of children developing CHB annually should reduce to about 0.5% (i.e.,101 CHB children in Nigeria) of the estimated present value in about two decades. However, in Nigeria, though HBV vaccine is administered at birth in tertiary health care facilities, the same cannot be said of secondary and primary health care facilities. Consequently, reduction in the number of children estimated to develop CHB to about 0.5% of the exposed may not have been achieved in the country. Though, the possibility of achieving the above stated still exist, since the country still has about a decade (WHO 2005a; Sadoh and Eregie 2008) to attain comparable situation with Taiwan (Su *et al.*^2012^) and Thailand (Poovorawan *et al.*^2011^).

Even if the National Immunization Programme in Nigeria achieves the reduction in estimated number of children developing CHB to 0.5% of the exposed, the presence of HBV IEMs and probable perinatal transmission to about 6.7% (one IEM out of 15 HBsAg positive; a lower limit of this study) of the perinatally exposed might undermine the impact of the vaccination effort by the immunization being ineffective in ~1347 children. This might result in the development of a cohort of CHB children that may serve as reservoirs for the maintenance of HBV, and specifically HBV IEMs in the population.

## Conclusion

The results of this study showed, for the first time in Nigeria, the presence of HBV isolates with mutations that have been associated with immune escape mutants. The results also confirmed circulation of HBV IEMs among pregnant women in Nigeria.
